# Virtual Reality in the acute psychiatry ward: a pilot study

**DOI:** 10.1192/j.eurpsy.2023.1910

**Published:** 2023-07-19

**Authors:** J. I. Mena, B. Andrés, I. Hernandez, A. Bastidas, E. Cesari, I. Ochandiano, S. Salmerón, P. Barrio

**Affiliations:** Psychiatry and Psychology Department, Hospital Clínic Barcelona, Barcelona, Spain

## Abstract

**Introduction:**

New technologies have been steadily impacting and redefining the health care landscape over the last decades, a process recently enhanced by the covid-19 pandemics . VR is an advanced media that can simulate highly realistic virtual environments, providing a high sense of immersion (the feeling of “being really there”). VR has expanded its healthcare application over the last years. Surprisingly, the acute psychiatry ward has been, so far, systematically left out of the VR application field. Psychiatric wards are complex environments. Patients are frequently admitted against their will and many wards have a locked doors policy, with subsequent feelings of seclusion experienced by patients. Therefore the question emerges: could VR help psychiatry inpatients have a better experience during their hospitalization?

**Objectives:**

This is a pilot study where psychiatry inpatients are offered a single session with the Oculus Quest 2, where they are immersed in a computer generate scenario provided by a commercially available software (“Nature Treks”). The scenario is a nature-based immersive 360° walk. Patients are allowed to freely explore the scenario with no time restraints.

**Methods:**

The STAI (State-Trait Anxiety Inventory), and the PANAS (Positive and Negative Affect Schedule) questionnaires are completed by patients before and after the VR exposure. After exposure, patients are also asked to complete the SUS (System Usability Scale) questionnaire, the IQ-presence questionnaire and the SSQ (Simulator Sickness Questionnaire). Electrophysiological recordings are gathered with the Empatica E4.

**Results:**

Up to date, 4 patients have been recruited. The sessions have lasted around 10 minutes. Reductions in the STAI and the PANAS have been reported by 3 patients (with no statistical significance so far). Usability has been extremely high as reported by the SUS. Minimal adverse reactions to VR use have been reported in the SSQ, mainly dizziness and nausea.

**Conclusions:**

VR has a high potential to ameliorate the conditions of psychiatry inpatients admitted to a close-doors ward. As with many technological novelties, implementation and sustainability will be key. The small evidence provided by this pilot study points out to an initial good acceptability and potential efficacy in some patient-related outcomes.

**Disclosure of Interest:**

None Declared

